# Probing the Phylogenomics and Putative Pathogenicity Genes of *Pythium insidiosum* by Oomycete Genome Analyses

**DOI:** 10.1038/s41598-018-22540-1

**Published:** 2018-03-07

**Authors:** Thidarat Rujirawat, Preecha Patumcharoenpol, Tassanee Lohnoo, Wanta Yingyong, Yothin Kumsang, Penpan Payattikul, Sithichoke Tangphatsornruang, Prapat Suriyaphol, Onrapak Reamtong, Gagan Garg, Weerayuth Kittichotirat, Theerapong Krajaejun

**Affiliations:** 1 Department of Pathology, Faculty of Medicine, Ramathibodi Hospital, Mahidol University, Bangkok, Thailand; 2 Research Center, Faculty of Medicine, Ramathibodi Hospital, Mahidol University, Bangkok, Thailand; 3 Molecular Medicine Program, Multidisciplinary Unit, Faculty of Science, Mahidol University, Bangkok, Thailand; 4 Department of Biomedical Informatics, University of Arkansas for Medical Sciences, Little Rock, Arkansas 72205 USA; 5 Systems Biology and Bioinformatics Research Group, Pilot Plant Development and Training Institute, King Mongkut’s University of Technology Thonburi, Bangkok, Thailand; 6 Genomic Research Laboratory, National Center for Genetic Engineering and Biotechnology, National Science and Technology Development Agency, Pathumthani, Thailand; 7 Bioinformatics and Data Management for Research, Office for Research and Development, Faculty of Medicine, Siriraj Hospital, Mahidol University, Bangkok, Thailand; 8 Department of Molecular Tropical Medicine and Genetics, Faculty of Tropical Medicine, Mahidol University, Bangkok, Thailand; 9 CSIRO Agriculture and Food, Centre for Environment and Life Sciences, Floreat, WA Australia

## Abstract

*Pythium insidiosum* is a human-pathogenic oomycete. Many patients infected with it lose organs or die. Toward the goal of developing improved treatment options, we want to understand how *Py*. *insidiosum* has evolved to become a successful human pathogen. Our approach here involved the use of comparative genomic and other analyses to identify genes with possible functions in the pathogenicity of *Py*. *insidiosum*. We generated an Oomycete Gene Table and used it to explore the genome contents and phylogenomic relationships of *Py*. *insidiosum* and 19 other oomycetes. Initial sequence analyses showed that *Py*. *insidiosum* is closely related to *Pythium* species that are not pathogenic to humans. Our analyses also indicated that the organism harbours secreted and adhesin-like proteins, which are absent from related species. Putative virulence proteins were identified by comparison to a set of known virulence genes. Among them is the urease Ure1, which is absent from humans and thus a potential diagnostic and therapeutic target. We used mass spectrometric data to successfully validate the expression of 30% of 14,962 predicted proteins and identify 15 body temperature (37 °C)-dependent proteins of *Py*. *insidiosum*. This work begins to unravel the determinants of pathogenicity of *Py*. *insidiosum*.

## Introduction

Oomycetes are a unique and evolutionarily diverse group of microorganisms that belong to the Kingdom Stramenopiles and share the microscopic hyphal morphology of fungi^1^. Based on phylogenetic analyses, oomycetes are closely related to diatoms and algae, and only distantly related to fungi^1,2^. Well-known oomycete genera include *Albugo*, *Aphanomyces*, *Bremia*, *Hyaloperonospora*, *Lagenidium*, *Peronospora*, *Phytophthora*, *Plasmopara*, *Pythium* and *Saprolegnia*^1,3–5^. Many oomycetes are pathogenic to economically important plants, while some species in the genera *Aphanomyces*, *Lagenidium*, *Pythium* and *Saprolegnia* are capable of infecting animals^1,5^. Only a few oomycetes (i.e. *Pythium insidiosum*^6^, *Pythium aphanidermatum*^7,8^ and *Lagenidium* species^9,10^) have been reported to cause devastating or fatal infections in humans. No commercially available drug is effective against the oomycete pathogens, making management of the infections caused by these organisms challenging.

Among the human-pathogenic oomycetes, *Py*. *insidiosum* (the causative agent of the life-threatening infectious condition called pythiosis) is the most frequently reported, with only four reported cases of infections with other species^6–11^. *Py*. *insidiosum* is also an invasive pathogen of nonhuman animals, such as horses, dogs, cats and cattle^11^. Available antifungal drugs are not useful in most cases of this infection, due to the organism’s lack of the drug-targeted enzymes for ergosterol biosynthesis^12^. Surgical removal of an infected organ is the main recourse to remove the infection, which often leads to permanent disabilities in surviving patients. Despite surgical intervention, many patients die from progressive and uncontrolled infection^6^. An effective treatment modality (i.e. anti-oomycete drug or vaccine) is thus urgently needed for the control of *Py*. *insidiosum* and related oomycetes.

A better understanding of the biology and pathogenesis of *Py*. *insidiosum* is required for the development of an efficient method for infection control. How *Py*. *insidiosum*, among the oomycetes, has evolved to become a successful human and animal pathogen is an open question that needs investigation. With the development of next-generation sequencing (NGS) technology, the genomes and transcriptomes of many oomycetes have been sequenced and are now publicly accessible^3,4,13–22^. Recently, we sequenced the first draft genome and transcriptome of *Py*. *insidiosum*, using the 454 and Illumina NGS platforms^19,20^. The genome of *Py*. *insidiosum* is 53.2 Mb in size and comprises 14,962 predicted open reading frames. This sequence information provides a useful resource for exploring the biology, evolution and pathogenicity of *Py*. *insidiosum*.

As a human and animal pathogen^11^, *Py*. *insidiosum* must have evolved a special set of genes that are required for this pathogenesis, which are not found in other oomycetes. In the current study, we analyse the genome content of *Py*. *insidiosum*, in comparison with that of 19 other oomycetes (including *Py*. *aphanidermatum*, which is a well-known plant pathogen, and sporadically causes human infections, including in two soldiers with severe blast injury in Afghanistan^7,8^) and 2 diatoms, to identify putative virulence genes, and shed light on the pathogenicity-related evolution of this pathogen. Since the ability to grow at the host’s body temperature is essential for an invasive pathogen, we also incorporated newly generated mass spectrometric data to validate and identify *Py*. *insidiosum* proteins that are differentially expressed in response to a shift in temperature from 25 °C to 37 °C. These studies have begun to reveal the factors behind the pathogenicity of *Py*. *insidiosum*.

## Results

### General characteristics of oomycete genomes

All genome sequences were retrieved from public databases (Table 1**)**. The oomycete genomes differ in size (33–229 Mb) and are larger than those of diatoms (26–31 Mb). These genomes have different G + C contents (43–59%), numbers of coding sequences (CDS; 11,958–27,941 genes) and CDS density (12–75%). *Albugo* and *Pythium* harbour smaller genomes (33–53 Mb) than other oomycetes (53–229 Mb). *Ph*. *infestans* has the largest genome (229 Mb) with the lowest CDS density (12%), whereas *Al*. *laibachii* has the smallest genome (33 Mb) with the highest CDS density (75%).

**Table 1:** Genomes of 20 oomycetes and 2 diatoms used in this study. Description of each genome includes species, strain, host specificity (plant, animal or human), genome size, contigs, N_50_, G + C content, coding sequences and data source. An asterisk indicates the two diatoms that serve as outgroups.

Species	Strain	Host	Genome size (Mb)	Number of contigs	N50 (bp)	G + C content (%)	Number of CDS	Average CDS (bp)	Total CDS length (bp)	CDS density (%)	Source
*Albugo laibachii*	Nc14	Plant	32.8	3,827	69,384	44.3	13,804	1,772	24,457,143	75	http://protists.ensembl.org
*Albugo candida*	2VRR	Plant	34.6	252	375,021	43.2	15,824	973	15,389,934	45	http://fungidb.org
*Hyaloperonospora arabidopsis*	Emoy2	Plant	78.9	3,044	332,402	47.2	14,321	1,105	15,826,807	20	http://protists.ensembl.org
*Phytophthora capsici*	LT1534	Plant	64.0	917	705,730	50.4	19,805	1,253	24,818,348	39	http://genome.jgi.doe.gov
*Phytophthora infestans*	T30–4	Plant	228.5	4,921	1,588,622	51.0	18,138	1,527	27,688,358	12	http://www.broadinstitute.org
*Phytophthora parasitica*	INRA-310	Plant	82.3	708	888,348	49.5	27,941	1,762	49,229,193	60	http://www.broadinstitute.org
*Phytophthora cinnamomi*	CBS 144.22	Plant	77.9	1,314	264,472	53.9	26,131	1,303	34,043,544	44	http://fungidb.org
*Phytophthora sojae*	P6497	Plant	86.0	1,810	462,795	54.4	16,989	1,618	27,494,907	32	http://www.broadinstitute.org
*Phytophthora ramorum*	pr102	Plant	66.6	2,576	308,042	53.9	14,394	1,629	23,440,930	35	http://www.broadinstitute.org
*Pythium vexans*	DAOM BR484	Plant	33.8	3,685	29,235	58.9	11,958	1,767	21,128,509	62	http://pythium.plantbiology.msu.edu
*Pythium irregulare*	DAOM BR486	Plant	42.9	5,887	23,217	53.8	13,805	1,707	23,568,848	55	http://pythium.plantbiology.msu.edu
*Pythium ultimum*	DAOM BR144	Plant	44.9	975	837,833	52.3	12,614	1,468	18,515,581	41	http://www.broadinstitute.org
*Pythium iwayamai*	DAOM BR242034	Plant	43.2	11,542	11,008	55.1	14,875	1,542	22,938,245	53	http://pythium.plantbiology.msu.edu
*Pythium arrhenomanes*	ATCC 12531	Plant	44.6	10,978	9,767	56.9	13,805	1,541	21,272,330	48	http://pythium.plantbiology.msu.edu
*Pythium aphanidermatum*	DAOM BR444	Human, Plant	35.8	1,774	37,384	53.8	12,312	1,618	19,925,113	56	http://pythium.plantbiology.msu.edu
*Pythium insidiosum*	Pi-S	Human, Animal	53.2	1,192	146,252	57.9	14,962	2,144	32,074,099	60	Accession: BBXB01000001–1192
*Aphanomyces astaci*	APO3	Animal	75.8	835	657,536	49.8	26,259	2,160	56,712,510	75	http://fungidb.org
*Aphanomyces invadans*	NJM9701	Animal	71.4	481	1,130,244	54.2	20,816	2,096	43,637,220	61	http://fungidb.org
*Saprolegnia declina*	VS20	Animal	62.8	390	602,571	58.6	18,229	1,779	32,427,679	52	http://www.broadinstitute.org
*Saprolegnia parasitica*	CBS 223.65	Animal	53.1	1,442	280,942	58.5	20,088	1,522	30,575,773	58	http://www.broadinstitute.org
Phaeodactylum tricornutum*	CCAP1055–1	—	26.1	33	945,026	48.9	10,025	1,621	16,248,565	62	http://genome.jgi-psf.org
Thalassiosira pseudonana*	CCMP1335	—	31.3	27	1,992,434	46.9	11,390	1,745	19,880,678	64	http://genome.jgi-psf.org

### Homologous gene cluster data

**Figure 1:**
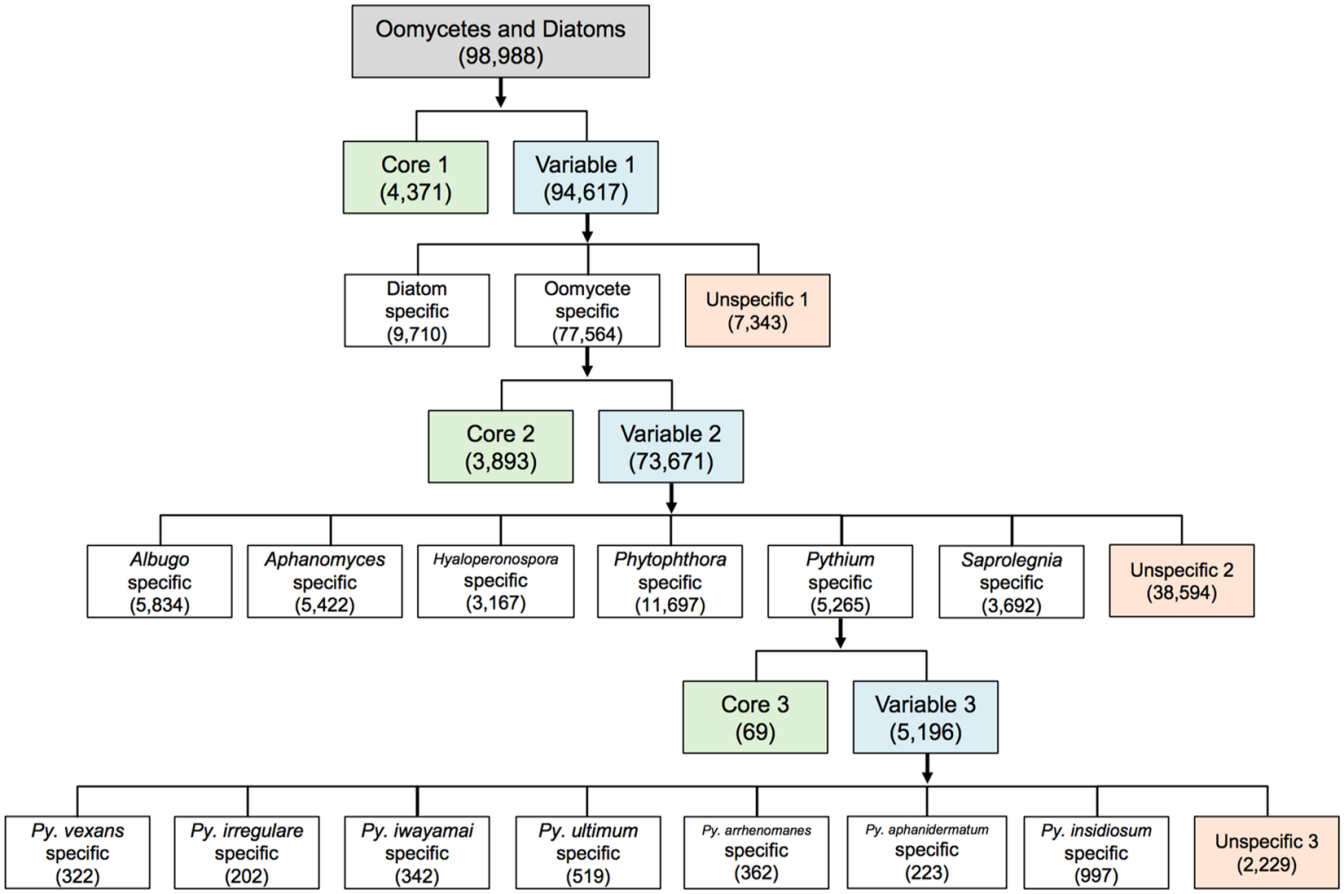
Flow chart of the 98,988 homologous gene clusters of the oomycetes and diatoms, selectively subgrouped into 2 major groups, 6 oomycete genera and 7 *Pythium* species. The ‘Core’ boxes (green) represent the shared gene clusters, identified in all major groups (assigned as Core 1), all oomycete genera (Core 2) and all *Pythium* species (Core 3). The ‘Variable’ boxes (blue) contain non-Core gene clusters, that is, Variable 1 (major groups), Variable 2 (oomycete genera) and Variable 3 (*Pythium* species). The ‘Unspecific’ boxes (orange) represent the gene clusters identified in at least two, if not all, major groups (assigned as Unspecific 1), oomycete genera (Unspecific 2) and *Pythium* species (Unspecific 3). The gene clusters (those specific to a particular major group, genus or species) are grouped together in the white boxes.

A total of 368,724 genes identified in 20 oomycete and 2 diatom genomes were grouped into 98,988 unique homologous gene clusters. The homologous gene cluster data used in this study can be assessed using our Oomycete Gene Table online tool, which can be found at http://www.sbi.kmutt.ac.th/cgi-bin/gt/viewer?organism=oomycetes&build=150418 (description and instructions of the Oomycete Gene Table will be reported in detail elsewhere). To narrow down the *Py*. *insidiosum*-specific gene clusters, we selectively classified these 98,988 gene clusters into subgroups based on gene content similarities, at the ‘major group’ level (including two groups, i.e. Diatoms and Oomycetes), the ‘genus’ level (including six oomycete genera, i.e. *Albugo*, *Aphanomyces*, *Hyaloperonospora*, *Phytophthora*, *Pythium* and *Saprolegnia*) and the ‘species’ level (including seven *Pythium* species, i.e. *Py*. *insidiosum*, *Py*. *irregulare*, *Py*. *iwayamai*, *Py*. *ultimum*, *Py*. *vexans*, *Py*. *arrhenomanes* and *Py*. *aphanidermatum*). Each ‘Core’ box (the green box in Fig. 1) represents the shared gene clusters, identified in (i) all 20 oomycetes and 2 diatoms (assigned as Core 1, containing 4,371 gene clusters), (ii) all 6 oomycete genera (assigned as Core 2, 3,893 gene clusters) and (iii) all 7 *Pythium* species (assigned as Core 3, 69 gene clusters). Each ‘Variable’ box (the blue box in Fig. 1) contains non-Core gene clusters, in each subgroup: Variable 1 (major groups, containing 94,617 gene clusters), Variable 2 (oomycete genera, 73,671 gene clusters) and Variable 3 (*Pythium* species, 5,196 gene clusters). Each ‘Unspecific’ box (the orange box in Fig. 1) represents the gene clusters, identified in at least two, but not all, major groups (assigned as Unspecific 1, containing 7,343 gene clusters), oomycete genera (assigned as Unspecific 2, 38,594 gene clusters) and *Pythium* species (assigned as Unspecific 3, 2,229 gene clusters). The gene clusters (those specific to a particular major group, genus or species) are grouped together (the white box in Fig. 1). *Py*. *insidiosum* contained a unique set of 997 gene clusters, not identified in other oomycete and diatom genomes.

BLAST searches against the Clusters of Orthologous Groups of Proteins (COGs) database^23,24^ demonstrated that 62% of the Core 1 gene clusters (belonging to oomycetes and diatoms) can be assigned to 22 out of 23 COG groups, with no gene matches for ‘Extracellular structures’ (Supplementary Fig. S1). Of the Core 2 gene clusters (belonging to the oomycetes), 23% can be allocated to 21 COG groups, with none in ‘Nuclear structure’ or ‘Extracellular structures’. Approximately 3% of the Core 3 gene clusters (belonging to the genus *Pythium*) had matches in two COG groups: ‘Cytoskeleton’ and ‘Post-translational modification, protein turnover, chaperones’.

### Gene content comparison

**Figure 2:**
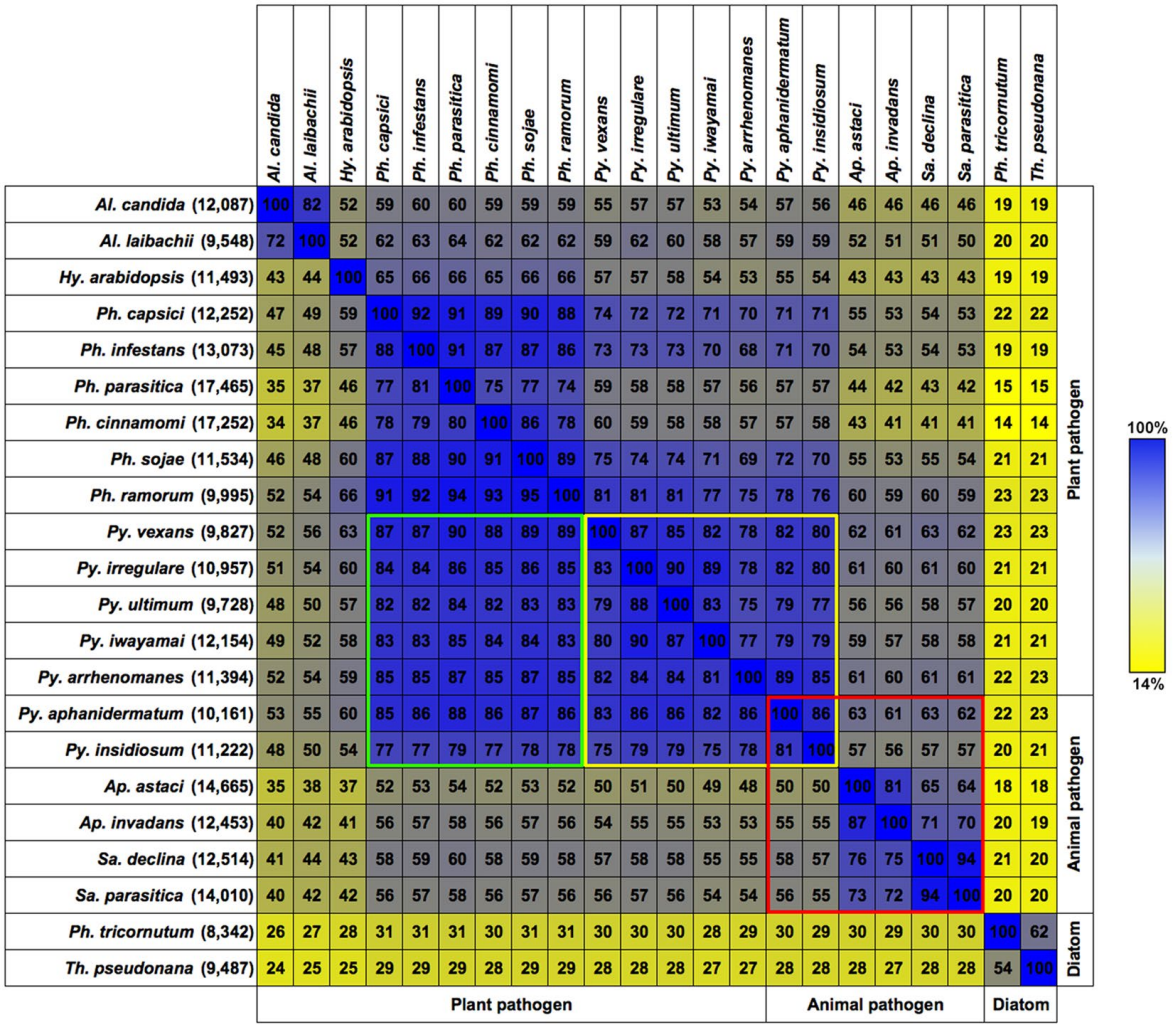
Pair-wise comparison of gene contents identified in 20 oomycete and 2 diatom genomes. The gene cluster number of each organism is shown in parentheses. The numbers and colour shadings indicate the percentage of the genes in each organism, listed on the left, that matched the genes in each organism, listed at the top. Pair-wise comparisons of the genera *Pythium* vs. *Phytophthora* (similarities: average, 84%; range, 77–90%) and the genera *Pythium* vs. *Pythium* (similarities: average, 82%; range, 75–90%) are depicted in the green and yellow boxes, respectively. Gene content similarities among six animal-pathogenic oomycetes (50–94%) are shown in the red box.

In Fig. 2, the gene content similarity is the proportion of the gene clusters identified in each organism, listed on the left of the figure, that matched the gene clusters identified in each organism, listed at the top. For example, the gene content similarity of *Py*. *insidiosum* (listed on the left of Fig. 2) vs. *Ph*. *infestans* (listed at the top) is 77%, indicating that 77% of the *Py*. *insidiosum* gene clusters matched those of *Ph*. *infestans*, whereas the gene content similarity of *Ph*. *infestans* (listed on the left of Fig. 2) vs. *Py*. *insidiosum* (listed at the top) is 70%, indicating that 70% of the *Ph*. *infestans* gene clusters matched those of *Py*. *insidiosum*. Less than 25% of gene clusters present in the oomycetes matched those of the diatoms. Within oomycetes of the same genus, there were at least 70% shared gene clusters, for example: *Albugo* (≥72%), *Phytophthora* (≥74%), *Pythium* (≥75%), *Aphanomycete* (≥81%) and *Saprolegnia* (≥94%). Among 6 animal-pathogenic oomycetes, there is a broad range of gene content similarities (50–94%; the red box in Fig. 2), as well as among 14 plant-pathogenic oomycetes (34–95%). When the pair-wise comparison was performed at the genus level, the genus *Pythium*, unlike other genera, demonstrated lower gene content similarities against itself on average (*Pythium* vs. *Pythium*; average, 82%; range, 75–90%; the yellow box in Fig. 2), compared with that against the genus *Phytophthora* (*Pythium* vs. *Phytophthora*; average, 84%; range, 77–90%; the green box in Fig. 2).

### Phylogenomic relationship

**Figure 3:**
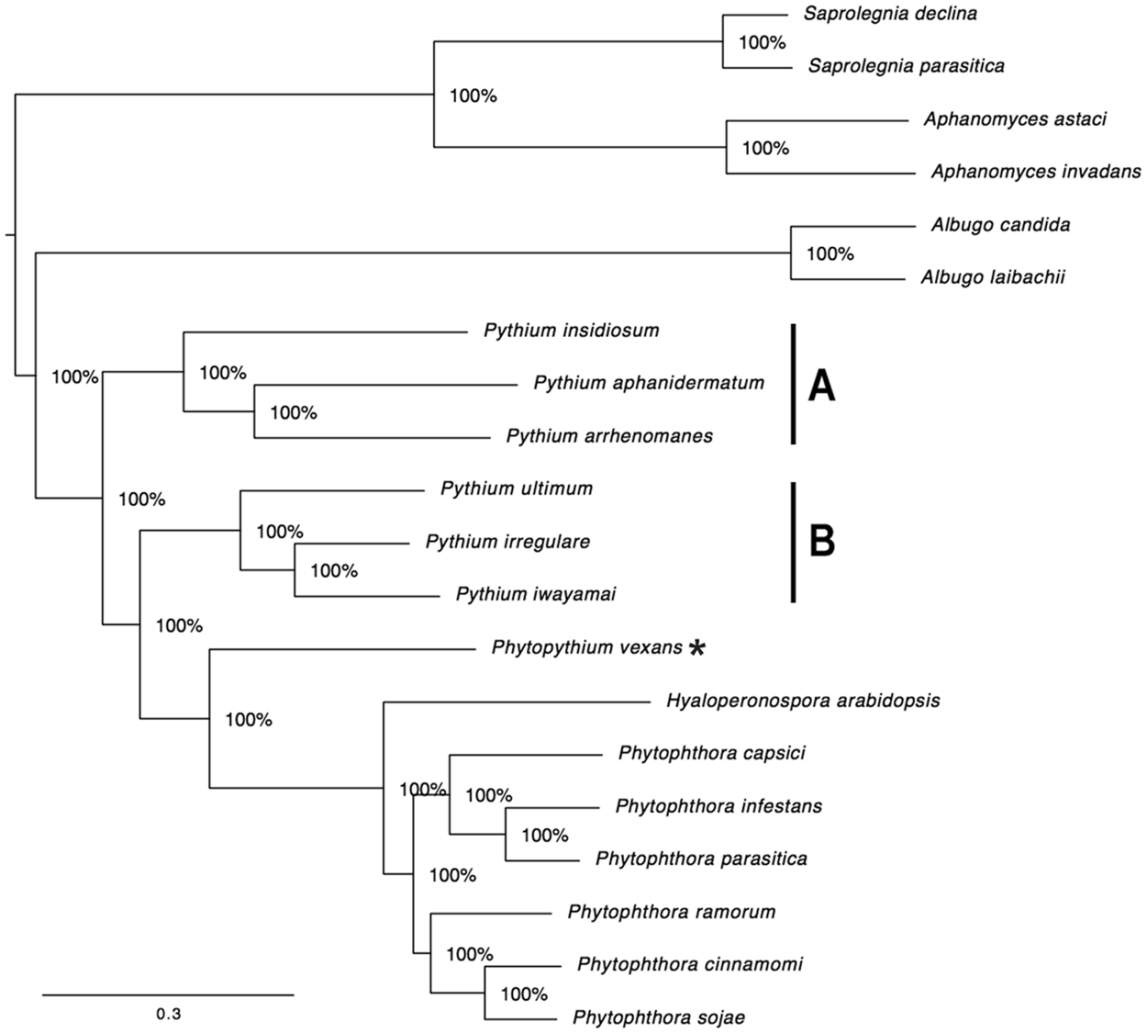
A maximum-likelihood phylogenomic tree generated by a set of 2,073 core genes found across all 20 oomycete genomes (total concatenated multiple sequence alignment length of 2,455,151). Descriptions of all 2,073 core genes are shown in Supplementary Table S1. The tree categorizes all *Pythium* species, except *Pythium vexans* (currently reclassified as *Phytopythium vexans*; indicated by asterisk), into clades **A** and **B**. The reliability of the trees tested by bootstrap analysis to support each branch is indicated.

To investigate the evolutionary relatedness and differences between oomycetes, a phylogenomic analysis was performed using a set of 2,073 core genes found across 20 oomycete genomes (Supplementary Table S1**)**. These core genes were found to produce phylogenetic trees with the best likelihood when the Generalized Time Reversal (GTR) evolutionary model was used (see the Methods). Concatenation of 2,073 individual core gene sequences produced a final multiple sequence alignment that is 2,455,151 positions in length. The concatenated multiple sequence alignment was then used to create a maximum likelihood phylogenomic tree using GTR as an evolutionary model. The resulting phylogenomic tree is shown in Fig. 3. Our phylogenomic tree separated almost all oomycetes according to their genera, with an exception to *Py*. *vexans*, which was separated from the other *Pythium* species, and phylogenetically proximal to the *Phytophthora* species and *Hy*. *arabidopsis*. For the genus *Pythium*, our phylogenomic analysis categorized all *Pythium* species, except *Py*. *vexans*, into two different clades: Group-A species (*Py*. *insidiosum*, *Py*. *aphanidermatum*, and *Py*. *arrhenomanes*), and Group-B species (*Py*. *irregulare*, *Py*. *iwayamai*, and *Py*. *ultimum*).

**Figure 4:**
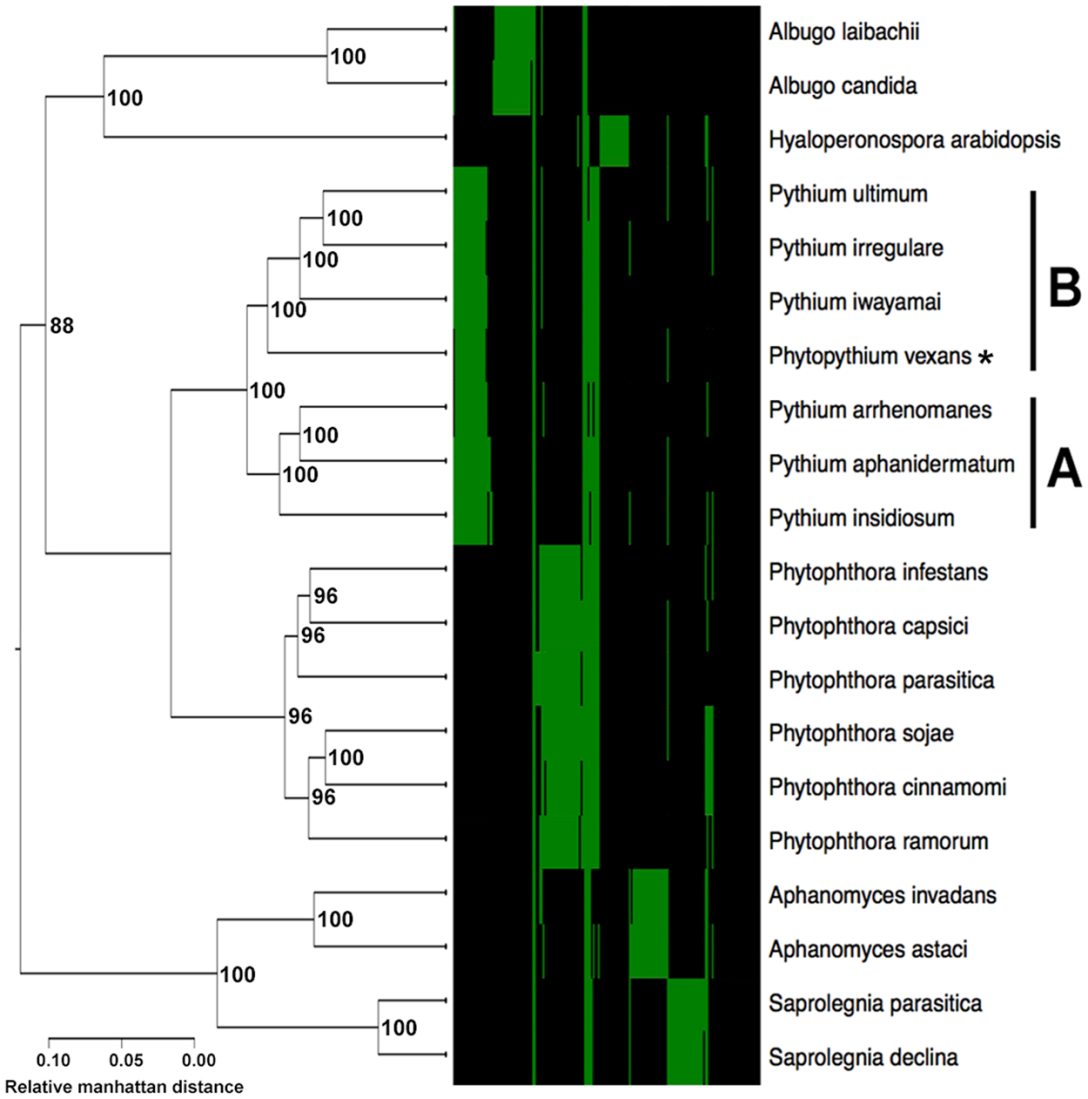
A phylogenomic tree based on hierarchical clustering of the gene presence profile of all 20 oomycetes. The heat map indicates the gene presence profile of 178 representative genes (green and black represent gene presence and absence, respectively). The tree categorises all *Pythium* species, including *Pythium vexans* (currently reclassified as *Phytopythium vexans*; indicated by an asterisk), into clades **A** and **B**. The reliability of the trees tested by bootstrap analysis to support each branch is indicated.

We also analyzed oomycete relatedness based on the hierarchical clustering of their gene presence profiles. In contrast to the 2,073 core gene-based phylogenomic tree, the resulting hierarchical clustering of gene presence profile-based tree showed that all oomycetes (including *Py*. *vexans*) were grouped according to their genera (Fig. 4**)**.

### Elicitin gene family in oomycetes

**Figure 5:**
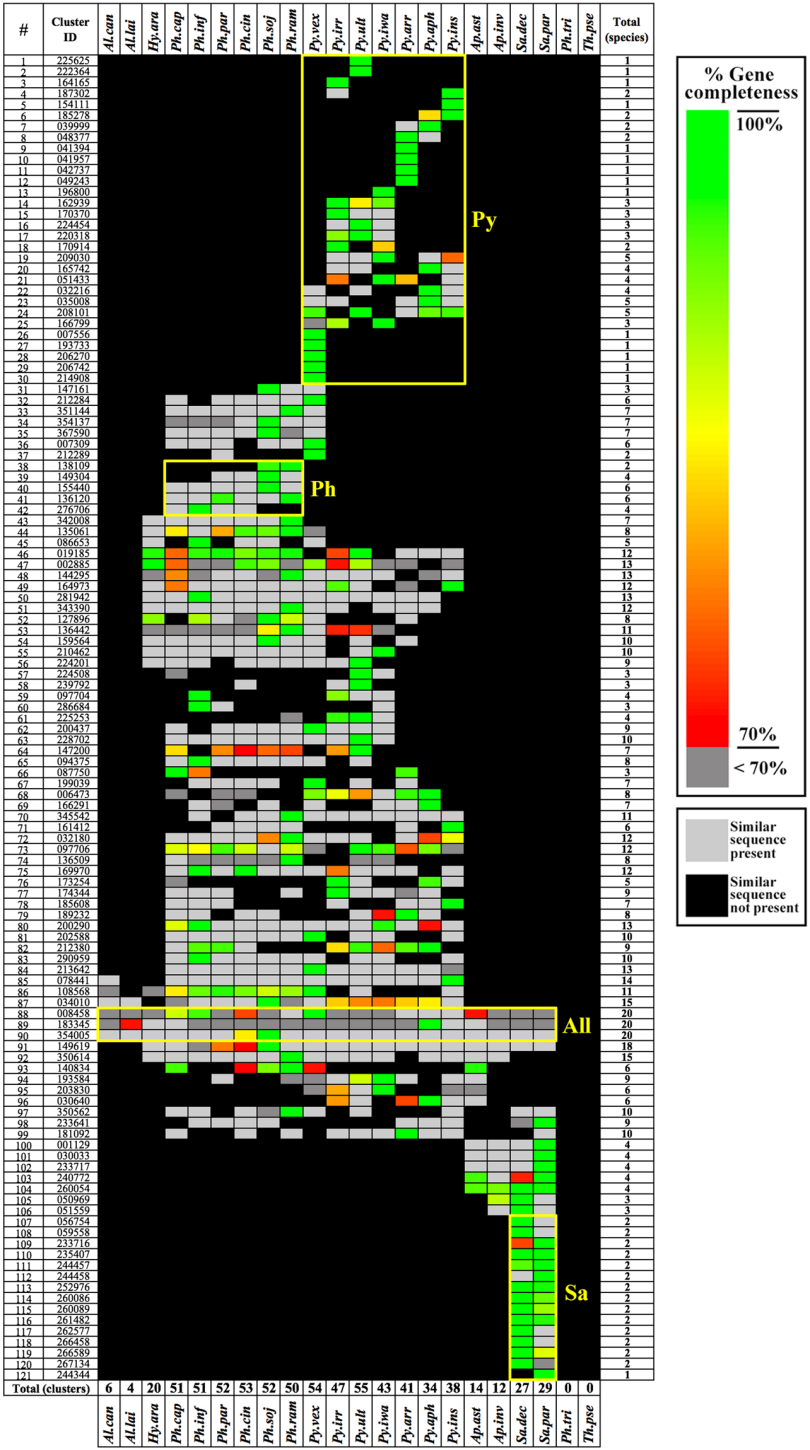
The Oomycete Gene Table shows a total of 121 elicitin gene clusters that are differently present in the oomycetes, but absent from the diatoms. The elicitin gene clusters 88, 89 and 90 are found across all oomycetes (as indicated by the ‘All’ box). Some elicitin gene clusters appear to be genus-specific: clusters 1–29 for the genus *Pythium* (the ‘Py’ box), 38–42 for the genus *Phytophthora* (the ‘Ph’ box) and 107–121 for the genus *Saprolegnia* (the ‘Sa’ box). The rightmost column shows the number of species that contain each of the elicitin gene clusters. The colour shadings correlate with percent completeness of each predicted gene, in relation to the longest gene of each gene cluster. The light grey box indicates the presence of a similar sequence (that does not fulfil our gene-defining criteria; see the Methods) and the black box indicates that neither a similar sequence nor gene is found.

Because of their association with pathogenicity, we were interested in investigating elicitin-encoding genes in the oomycete genomes. A total of 121 elicitin gene clusters, defined by the Oomycete Gene Table, were differentially present in all oomycetes, but absent in diatoms (Fig. 5). The organisms in the genera *Pythium* and *Phytophthora* harboured more elicitin gene clusters (average, 48 clusters/species; range, 34–55) than other genera (average, 13 clusters/species; range, 4–29). The elicitin gene clusters 88, 89 and 90 were found across all oomycetes (the ‘All’ box in Fig. 5). Some elicitin gene clusters appeared to be genus-specific, that is, clusters 1–29 of *Pythium* (the ‘Py’ box in Fig. 5), 38–42 of *Phytophthora* (the ‘Ph’ box) and 107–121 of *Saprolegnia* (the ‘Sa’ box).

### Genome annotation of *Py*. *insidiosum*

BLAST searches of the 14,962 predicted proteins of *Py*. *insidiosum* against the NCBI database showed that 2,811 proteins (19%) matched proteins with known functions, 10,606 (71%) matched hypothetical proteins and 1,545 (10%) had no significant sequence similarity. BLAST2GO assigned 1,405 Gene Ontology (GO) terms to 7,822 proteins (52% of all proteins) in three categories: (i) 550 GO terms in the Biological Processes (4,404 proteins), (ii) 162 GO terms in the Cellular Components (2,261 proteins) and (iii) 693 GO terms in the Molecular Functions (6,946 proteins). The top 40 GO terms assigned in each category are shown in Supplementary Figure S2. BLAST2GO also allocated 1,177 proteins (8% of all proteins) with 1,296 different enzyme commission (EC) numbers into six classes: Oxidoreductases (193 proteins), Transferases (442 proteins), Hydrolases (425 proteins), Lyases (68 proteins), Isomerases (65 proteins) and Ligases (103 proteins) (Supplementary Table S2). Based on InterProScan, 5,113 different domains were detected in 10,242 proteins (68% of all proteins) and the top 40 domains are shown in Supplementary Table S3. SignalP identified signal peptides (average length, 23 amino acids; range, 11–56) in 1,208 proteins (8% of all proteins). Transmembrane helices were predicted in 3,142 proteins (21% of all proteins).

### Validation of predicted proteins of *Py*. *insidiosum*

*Py*. *insidiosum* protein extracts (SABHs) were prepared in four replicates, two each from the organism grown at either 25 °C or 37 °C (designated as 25 °C and 37 °C SABHs, respectively), and subjected to LC-MS/MS analysis. The set of 14,962 predicted proteins of *Py*. *insidiosum*, theoretically digested with trypsin, was used as an in-house Mascot library for LC-MS/MS search. Any peptide identified by LC-MS/MS in at least two replicates was recruited for protein validation, resulting in a pool of 120,265 peptides. A total of 4,445 genome-derived proteins (30% of all proteins), mapped with at least two different recruited peptides, were validated for their actual expression in *Py*. *insidiosum*. The average number of mapped peptides per protein was 27 (range, 2–200).

### Effect of temperature on protein expression in *Py*. *insidiosum*

Changes in *Py*. *insidiosum* protein abundance, upon exposure to increased temperature, were assessed using LC-MS/MS data of the 25 °C and 37 °C SABHs (two replicates each). Unquantifiable proteins, assigned as ‘no emPAI’ (see the Methods), were excluded. There were 344 validated proteins recruited for comparison of protein abundances. *Py*. *insidiosum* exhibited 212 relatively high- and 124 relatively low-abundance proteins in the 37 °C SABHs, compared with the 25 °C SABHs. Of these differentially expressed proteins, 53 and 40 were at least twofold up- and downregulated, respectively (Supplementary Table S4). The top upregulated proteins included cyclophilin-A (45-fold increase), peroxiredoxin-2 (15-fold), inositol-3-phosphate synthase (13-fold), alanine aminopeptidase (10-fold) and proteasome subunit beta type-6 (10-fold), whereas the markedly downregulated ones included 6-phosphogluconate dehydrogenase (40-fold decrease), a hypothetical protein (22-fold) and glucokinase (10-fold).

### *Py*. *insidiosum*-specific genes

Based on the Oomycete Gene Table, which included 98,988 unique homologous gene clusters, 997 gene clusters were found only in *Py*. *insidiosum* (Fig. 1). From these *Py*. *insidiosum*-specific gene clusters, we identified 1,194 predicted genes. Of these, 85% (1,013 predicted proteins) had no positive BLAST hits in the NCBI database and the other 15% (181 proteins) matched proteins with unknown function. The LC-MS/MS analyses detected peptides that map to 116 of these *Py*. *insidiosum*-specific proteins. Signal peptide and transmembrane helices were predicted in 194 and 199 proteins, respectively. Thirty-three adhesin-like proteins were predicted by FungalRV.

### Putative virulence genes of *Py*. *insidiosum*

All predicted proteins were used as queries for a BLAST search against the MvirDB database, a collection of pathogen virulence factors^25^. A total of 1,820 proteins (12% of all proteins) found matches, which can be classified into five groups: (i) virulence factors (887 proteins), (ii) pathogenicity islands (584 proteins), (iii) toxins (240 proteins), (iv) antibiotic resistance-related proteins (99 proteins) and (v) a miscellaneous group (10 proteins). The top 40 protein hits for the defined virulence factors are listed in Table 2. The matched virulence factors with highest probability (*E*-value, 0.0) were heat shock protein 70, carbamoyl phosphate synthetase II, chaperone protein ClpB, oligopeptidase B, tripeptidyl peptidase 2 and urease.

**Table 2:** Forty top-ranking putative virulence proteins of *Pythium insidiosum*, based on sequence similarity and probability (*E*-value), obtained by a BLAST search against the MvirDB database (see the Methods). The proteins that can be mapped with at least two LC-MS/MS-generated peptides are indicated in the last column.

Protein ID	VFID	Virulence factor type	Description	Organism	Identities (%)	E-value	Number of mapped peptides
PINS01090021A	26447	Virulence protein	Heat shock protein 70	*Toxoplasma gondii*	76.4	0.0E + 00	12
PINS03900001A	26447	Virulence protein	Heat shock protein 70	*Toxoplasma gondii*	76.2	0.0E + 00	—
PINS00530003B	26381	Virulence protein	Heat shock protein 70	*Cryptosporidium parvum*	71.2	0.0E + 00	82
PINS01800019A	26447	Virulence protein	Heat shock protein 70	*Toxoplasma gondii*	66.2	0.0E + 00	6
PINS01150027A	26468	Virulence protein	Heat shock protein 70	*Eimeria tenella*	65.0	0.0E + 00	37
PINS00920015C	12025	Virulence protein	Urease	*Oryza sativa*	64.5	0.0E + 00	40
PINS01090006A	26447	Virulence protein	Heat shock protein 70	*Toxoplasma gondii*	61.2	0.0E + 00	—
PINS00020096A	26447	Virulence protein	Heat shock protein 70	*Toxoplasma gondii*	57.6	0.0E + 00	20
PINS02610001A	26447	Virulence protein	Heat shock protein 70	*Toxoplasma gondii*	57.6	0.0E + 00	—
PINS01620006A	15128	Virulence protein	Chaperone protein ClpB	*Francisella tularensis*	55.1	0.0E + 00	32
PINS04750002A	26434	Virulence protein	Carbamoyl phosphate synthetase II	*Toxoplasma gondii*	53.3	0.0E + 00	30
PINS02520008A	26434	Virulence protein	Carbamoyl phosphate synthetase II	*Toxoplasma gondii*	51.5	0.0E + 00	24
PINS00060002C	26434	Virulence protein	Carbamoyl phosphate synthetase II	*Toxoplasma gondii*	48.0	0.0E + 00	102
PINS00250041A	27174	Virulence protein	Oligopeptidase B	*Trypanosoma brucei brucei*	47.4	0.0E + 00	4
PINS00320055A	15128	Virulence protein	Chaperone protein ClpB	*Francisella tularensis*	47.1	0.0E + 00	16
PINS01420022C	11169	Protein toxin	Tripeptidyl peptidase 2	*Mus musculus*	34.8	0.0E + 00	24
PINS05940001B	26381	Virulence protein	Heat shock protein 70	*Cryptosporidium parvum*	73.5	1.0E-177	17
PINS00130063A	26455	Virulence protein	Eukaryotic translation initiation factor 4 A	*Toxoplasma gondii*	72.9	1.0E-173	31
PINS00020065A	8350	Virulence protein	Chaperonin GroEL	*Legionella pneumophila*	57.0	3.0E-159	61
PINS00550026A	8728	Virulence protein	Phosphoglucomutase	*Brucella melitensis*	50.6	2.0E-158	50
PINS00550030A	8728	Virulence protein	Phosphoglucomutase	*Brucella melitensis*	50.4	7.0E-158	80
PINS01120025A	26459	Virulence protein	Peroxisomal catalase	*Toxoplasma gondii*	59.6	9.0E-157	8
PINS01120010A	26459	Virulence protein	Peroxisomal catalase	*Toxoplasma gondii*	59.5	3.0E-156	—
PINS01900015C	12413	Protein toxin	Transcription factor site-1 protease	*Homo sapiens*	41.6	2.0E-155	—
PINS00150050B	20164	Pathogenicity island	Asparaginyl-tRNA synthetase	*Salmonella enterica*	58.0	1.0E-153	25
PINS04900006A	26455	Virulence protein	Eukaryotic translation initiation factor 4 A	*Toxoplasma gondii*	64.3	7.0E-152	—
PINS00400067B	26455	Virulence protein	Eukaryotic translation initiation factor 4 A	*Toxoplasma gondii*	64.6	8.0E-151	—
PINS01020032C	27223	Virulence protein	80 kDa prolyl oligopeptidase	*Trypanosoma cruzi*	41.3	1.0E-147	29
PINS00400051A	26455	Virulence protein	Eukaryotic translation initiation factor 4 A	*Toxoplasma gondii*	63.0	7.0E-146	—
PINS00050027A	19089	Pathogenicity island	Methylmalonyll-CoA mutase	*Streptomyces coelicolor*	50.3	4.0E-145	—
PINS02120014C	12413	Protein toxin	Transcription factor site-1 protease	*Homo sapiens*	40.2	5.0E-142	30
PINS00780045C	15600	Pathogenicity island	GDP-mannose 4,6-dehydratase	*Bradyrhizobium japonicum*	66.2	4.0E-139	17
PINS07960001C	15600	Pathogenicity island	GDP-mannose 4,6-dehydratase	*Bradyrhizobium japonicum*	63.8	8.0E-137	9
PINS01820002A	18499	Pathogenicity island	CTP synthetase	*Streptococcus agalactiae*	44.7	3.0E-125	36
PINS00650054A	26447	Virulence protein	Heat shock protein 70	*Toxoplasma gondii*	42.8	2.0E-121	—
PINS01020025B	7573	Virulence protein	Nonribosomal peptide synthetase DhbF	*Bacillus anthracis*	29.0	1.0E-113	54
PINS01050006C	18594	Pathogenicity island	Polynucleotide phosphorylase/polyadenylase	*Streptococcus agalactiae*	37.7	2.0E-113	—
PINS01020014C	7573	Virulence protein	Nonribosomal peptide synthetase DhbF	*Bacillus anthracis*	28.9	3.0E-113	28
PINS00170005A	20164	Pathogenicity island	Asparaginyl-tRNA synthetase	*Salmonella enterica*	46.7	6.0E-113	27
PINS00080003C	28065	Virulence protein	Dynamin-like protein	*Giardia intestinalis*	43.7	1.0E-110	11

**Figure 6:**
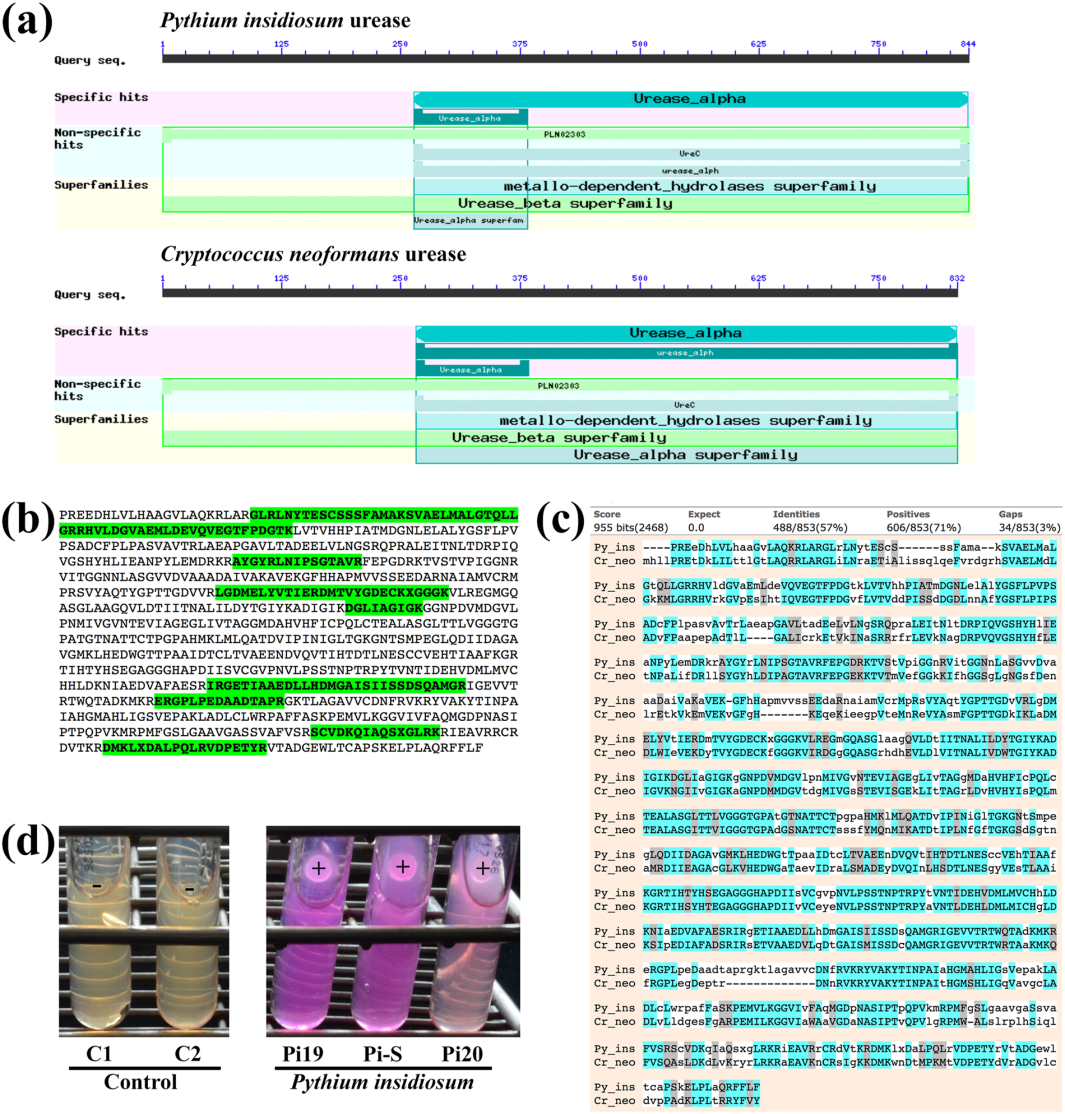
Characterisation of the *Pythium insidiosum* urease protein Ure1: (**a**) Comparison of urease domain architecture of *Py*. *insidiosum* Ure1 and *Cr*. *neoformans* URE1 (the bars indicate the length of each protein). (**b**) LC-MS/MS-derived peptides map eight different regions in the Ure1 sequence. (**c**) Two-sequence alignment of the Ure1 and the *Cryptococcus neoformans* urease URE1 (accession number, AAC62257). (**d**) Results of the urease test of three different *Py*. *insidiosum* isolates (i.e. Pi19, Pi-S and Pi20; the test is read as positive (+) if the colour turns pink and negative (−) if it remains yellow; the controls C1 and C2 were tested with an agar plug without the growing organism).

Among the top-ranking putative virulence proteins of *Py*. *insidiosum* (Table 2**)**, the urease (Ure1), which is 844 amino acids long (Fig. 6a), was selected for further characterisation. Forty LC-MS/MS peptides map to eight different regions in the Ure1 sequence (Fig. 6b). The *Py*. *insidiosum* urease, Ure1, had a high degree of sequence homology (*E*-value, 0.0; identity, 57%; similarity, 71%; Fig. 6c) and shared domain architecture (Fig. 6a) with the *Cryptococcus neoformans* urease URE1 (accession number, AAC62257). Three different clinical isolates (Pi19, Pi20 and Pi-S) of *Py*. *insidiosum* gave a positive urease reaction (Fig. 6d).

## Discussion

The genomes of 20 oomycetes and 2 diatoms altogether contained 368,724 genes, which can be grouped into 98,988 homologous gene clusters (Fig. 1). Of all of the unique gene clusters, 4,371 were assigned to ‘Core 1’ as they were found in every genome. However, since the genome data of *Py*. *arrhenomanes* and *Py*. *iwayamai* appeared to have lower quality (i.e. a larger number of contigs and shorter N_50_ length; Table 1), this number of core gene clusters was probably lower than it actually was. The core gene clusters (i.e., Cores 1, 2 and 3) generally contained housekeeping genes, whereas most of the other gene clusters contained genes with no defined function (Fig. 1 and Supplementary Fig. S1). Pair-wise comparison of the gene contents, at the genus level, showed an unexpected finding, where the similarities of *Pythium* vs. *Pythium* (average, 82%) were lower than those of *Pythium* vs. *Phytophthora* (average, 84%) (Fig. 2). This finding suggests that the vast majority of gene contents present in the genus *Pythium* are present in the genus *Phytophthora* (which generally harbours larger genomes than *Pythium*; average genome size: 101 Mb vs. 43 Mb; Table 1). How evolution contributed to this paradoxical observation in *Phytophthora* and *Pythium* is an interesting issue, and requires further investigation.

We explored the evolutionary relationships among oomycetes, at the genome scale, by generating hierarchical clustering of the gene presence profile-based tree (which summarises the relatedness of genomes according to the gene contents) and a 2,073 core gene-based tree (which focuses on the relatedness of genomes according to the core gene set). The former tree, based on the gene content, showed that all oomycete organisms were nicely grouped according to their genera (Fig. 4). However, the latter tree, based on the core genes, placed the organisms into expected clades, according to their genera, with an exception for *Py*. *vexans* (which appeared in a separate clade, apart from *Pythium*, but closer to the *Phytophthora* species and *Hy*. *arabidopsis*) (Fig. 3). Recently, *Py*. *vexans* has been reclassified into the genus ‘*Phytopythium*’, and its current binomial name is *Phytopythium vexans*, based on phylogenomic analyses^26^. Taken together, our results suggest that species of *Pythium* and *Phytopythium* share a common ancestor, but may have secondarily gained or lost different sets of genes as they went through their evolutionary journey. For the genus *Pythium*, our phylogenetic trees separated various *Pythium* species into two different clades (Figs 3 and 4), which were correlated with their pathogenic characteristics. Specifically, two of the Group A species (i.e. *Py*. *insidiosum* and *Py*. *aphanidermatum*) are known to have the ability to infect humans, whereas all Group B species are strictly plant pathogens^6–8,11^.

Elicitins form a unique protein family found in oomycetes (mainly *Phytophthora* and *Pythium*) and are not present in other organisms^27–29^. The proteins function as sterol carriers and act as ‘pathogen-associated molecular patterns’ in plants^30–34^. We used the Oomycete Gene Table to search for elicitins in 22 genomes (Table 1). Elicitins were absent from diatoms, but present in all oomycetes (Fig. 5). *Phytophthora* and *Pythium* harboured a more extensive set of elicitins than the other genera. The elicitin family, thus, could be an example of genes that existed in a common ancestor and subsequently underwent differential gene expansion, gene loss and gene modification during evolution.

There are 14,962 predicted genes in the genome of *Py*. *insidiosum*^19^, most of which (~80%) had no match to any given genes with defined function. Predicting protein sequences based on predicted eukaryotic genes, especially those without homologous sequences in the database, can be complicated by the presence of introns and six possible reading frames. Peptide mapping can circumvent this obstacle, and it enabled us to successfully validate ~30% of the theoretically translated proteins of *Py*. *insidiosum*. Peptide mapping is thus a useful method that can increase the reliability of gene annotation. We also utilised the mass spectrometric data to initially explore proteins that might be associated with the growth of *Py*. *insidiosum* at body temperature. Fifteen proteins exhibited ≥5-fold greater abundance upon a shift to a higher temperature (25 °C to 37 °C; Supplementary Table S4). The SignalP program^35^ identified no signal peptide among these 15 upregulated proteins, suggesting that they are nonsecreted proteins and could function inside the cell. In addition, based on the COG analysis^23,24^, the majority of these proteins were predicted to be involved in: (i) metabolism and transportation of amino acids, nucleotides, carbohydrates or lipids (eight proteins); and (ii) post-translational modification, protein turnover and chaperones [five proteins, including cyclophilin-A, which was the most highly (45-fold) upregulated]. Such upregulated proteins might prove to be necessary for the temperature tolerance and infectivity of *Py*. *insidiosum*.

Most pathogenic oomycetes infect plants. *Py*. *aphanidermiatum*, a well-known plant pathogen, has been recently reported to infect humans^7,8^. Unlike *Py*. *insidiosum*, the human infection caused by *Py*. *aphanidermatum* is extremely rare (only two cases) and has only been reported in patients with an unusual status (soldiers with severe blast injury), living in a specific geographic location (i.e. Afghanistan). Differences in host range, underlying status of the host and clinical features suggest different mechanisms behind the pathogenicity of *Py*. *insidiosum* and *Py*. *aphanidermatum*. In the current study, we focus on how *Py*. *insidiosum* developed the ability to infect humans. It would be expected that *Py*. *insidiosum* has gained a special set of genes necessary for human infection during its evolution. We began to address this hypothesis by looking for *Py*. *insidiosum*-specific genes (based on comparative genomic analysis) and putative virulence factors (based on an MvirDB database search)^25^. We found 1,194 *Py*. *insidiosum*-specific proteins, without homology to any reported proteins. Of these proteins, ~10% can be readily validated (by peptide mapping) in terms of their expression in *Py*. *insidiosum*. Secretory and surface proteins are important for microbial pathogenesis, as they act at the interface of host–pathogen interactions. A number of secretory and adhesin-like proteins (~200 each) were predicted, and could serve as a subset of virulence factor candidates, to be the subject of further analysis. The life cycle of *Py*. *insidiosum* has already been reported by Mendoza and colleagues^36^. Zoospores are considered to be infective units that could initiate infection, upon direct exposure to humans or other animals. In conjunction with the genome study, future transcriptome analysis of the zoospores could lead to the identification of genes responsible for the initial stage of *Py*. *insidisoum* infection.

Different pathogens may employ similar pathogenic strategies, using shared virulence factors. *Py*. *insidiosum* contained 1,820 proteins that matched known virulence factors^25^. Among the top-ranking putative virulence proteins of *Py*. *insidiosum* (*E*-value, 0.0; Table 2), only the urease (Ure1) was unmatched in the NCBI reference proteins of humans (taxid: 9606), suggesting Ure1 as a potential diagnostic and therapeutic target. The LC-MS/MS-derived peptides mapped throughout the Ure1 sequence (Table 2**;** Fig. 6b), indicating that Ure1 was actually expressed. Because *Py*. *insidiosum* had one copy of the urease-encoding gene (*Ure*1) in its genome, the positive result of the urease test (Fig. 6d) was probably due to urease activity of the Ure1 protein. In pathogenic bacteria and fungi (i.e. *Helicobacter pylori* and *Cr*. *neoformans*), urease hydrolyses host urea to ammonia, in order to counteract the acidic environment, inhibit immune responses or destroy host tissue^37–39^. The *Py*. *insidiosum* urease Ure1 protein shares significant sequence similarity and domain architecture with the *Cr*. *neoformans* URE1 (Fig. 6a,c). Functional characterisation of Ure1 (i.e. gene cloning and expression, loss-of-function study by gene silencing and pathogenicity study in an animal model) is underway to investigate whether this enzyme is involved in the pathogenesis of *Py*. *insidiosum*.

In summary, we generated an Oomycete Gene Table to explore the genome contents and phylogenomic relationships of *Py*. *insidiosum* and 19 other oomycetes. Changes in gene contents, during the course of evolution, could have contributed to phenotypes that might be associated with speciation, host specificity and virulence of the oomycetes. *Py*. *insidiosum* is a prominent human pathogenic oomycete that appeared to be closely related to *Pythium* species that are not pathogenic to humans. The human specificity of *Py*. *insidiosum* should depend on a set of acquired pathogenicity genes. Indeed, a handful of *Py*. *insidiosum*-specific, body temperature-dependent and putative virulence proteins have been identified, and could serve as initial targets for investigating the mechanisms behind the pathogenesis of this species. The *Py*. *insidiosum* urease Ure1 is a putative virulence factor, as it shared sequence similarity and domain architecture with the virulence factor urease, URE1, of *Cr*. *neoformans*. Since humans do not contain urease^39^, Ure1 is a potential diagnostic and therapeutic target of *Py*. *insidiosum*.

## Methods

### Ethics statement

This study was approved by the Committee on Human Rights Related to Research Involving Human Subjects, at the Faculty of Medicine, Ramathibodi Hospital, Mahidol University (approval number MURA2011/412). All methods were performed in accordance with the relevant guidelines and regulations.

### Genome sequences of *Py*. *insidiosum* and related oomycetes

The genome sequences of *Py*. *insidiosum* (strain Pi-S) and 19 other oomycetes, including 6 *Pythium* species, 6 *Phytophthora* species, 2 *Albugo* species, 2 *Aphanomyces* species, 2 *Saprolegnia* species and a *Hyaloperonospora* species, were retrieved from public databases (Table 1). Genome sequences of two diatoms were also recruited to serve as outgroups (Table 1).

### Grouping of genes into homologous gene clusters for gene content comparison

All genes found in the 22 genomes (Table 1) were processed in a sequence similarity-based gene grouping to compare gene content across multiple genomes using a Gene Table protocol, previously published by Kittichotirat and colleagues^40^. For each sequence comparison, we used the following thresholds to group genes into the same cluster: BLAST *E*-value of 10^−6^, pairwise sequence identity of at least 30% and pairwise sequence alignment coverage for both query and subject of at least 50%. The final homologous gene cluster results are presented in the Oomycete Gene Table, where each row represents a gene and each column represents a genome used in this study. Each cell in the table contains information regarding homologous genes or genomic regions that were found in the corresponding genome.

### Identification and alignment of core genes across oomycete genomes

Core gene were identified by scanning through the homologous gene cluster data for genes that appeared to be present in all 20 oomycete genomes. For each core homologous gene cluster, the longest gene member was used as a query and orthologous sequences from other oomycete genomes were identified by using NCBI TBLASTN^41^ with e-value cutoff of 1e-6 and coverage of more than 50%. We obtained orthologous core gene sequences in this manner to avoid cases where a gene was not originally identified in a genome even though the sequence or a part of the sequence is present in the genome sequence data. The orthologous sequences of each gene were then aligned using the PhylomeDB pipeline (https://github.com/Gabaldonlab/phylomizer)^42^. Briefly, three different software, which are MUSCLE v3.7^43^, MAFFT v7.313^44^ and Kalign v2.04^45^ were used to create multiple sequence alignments in both forward and reverse directions (also known as Head or Tail approach^46^). M-Coffee^47^ was then used to combine all six multiple sequence alignment results into a consensus alignment. Finally, trimAl v1.4rev22^48^ was used to trim poorly aligned regions present in the consensus alignment result with the consistency and gap score cut-off of 0.1667 and 0.1 respectively.

### Reconstruction of oomycete phylogenomic tree

The phylogenomic tree reconstruction was done by using the approach described by Capella-Gutierrez and co-workers^49^. Briefly, the process started by reconstructing a phylogenetic tree from each alignment using the Neighbor Joining (NJ) method^50^. Seven different evolutionary models (HKY85, JC69, K80, F81, F84, TN93, and GTR) were then used to calculate the likelihood of the NJ tree topology, with branch-length optimization permitted, using PhyML v3.0^51^. Next, the AIC criterion^52^ was used to compare the likelihood of the used models to identify the model that fitted best with each alignment data. This process was applied to all core gene alignments and the alignments were grouped based on the best fitted evolutionary model. The individual alignments in the largest group were then concatenated and PhyML v3.0 was used to construct the Maximum Likelihood tree from the concatenated alignment using the corresponding best fitted model with the following options: (i) Tree topology search operation set to Subtree Pruning and Regrafting (SPR), (ii) Nucleotide frequencies estimated by counting the occurrence of the different bases in the alignment, (iii) Proportion of invariable sites estimated by the maximum likelihood approach, (iv) Value of the gamma shape parameter estimated by the maximum likelihood approach, (v) Number of relative substitution rate categories set to 8, (vi) Branch supports set to Chi2-based parametric method, (vii) Parameter optimizations carried out on tree topology, branch length and substitution rate parameters. Finally, the FigTree software v1.4.0 (http://tree.bio.ed.ac.uk/software/figtree/) was used to draw the phylogenomic tree result where tree rooting was done using midpoint approach.

### Genome relatedness based on hierarchical clustering of gene presence profile

The homologous gene cluster result was used to create the gene presence profile data. A value of 0 or 1 was assigned to denote the absence or presence of a gene in each genome, respectively. A value of 1 was also assigned to cases in which a gene was not predicted but a homologous genomic region was found in that particular genome. Hierarchical clustering of gene presence profiles based on relative Manhattan distance and bootstrapping analysis was performed using the R programming language.

### Annotation of *Py*. *insidiosum* genome

The 53.2-Mb draft genome sequence of *Py*. *insidiosum* (generated from the Illumina HiSeq. 200 and 454 FLX Titanium NGS platforms)^19^ underwent gene prediction, using the transcriptome of *Py*. *insidiosum*^20^, the predicted proteome of *Py*. *ultimum*^18^ and an array of bioinformatics tools^53^, including RepeatMasker (http://www.repeatmasker.org), CEGMA 2.5^54^, GeneMark-ES^55^, Augustus 2.5.5^56^ and MAKER2^57^. To annotate the genome of *Py*. *insidiosum*, all deduced protein sequences were subjected to (i) a BLAST search against the NCBI nonredundant protein database (*E*-value cut-off, <−6), (ii) GO assignment using BLAST2GO^58^ and (iii) domain identification using InterProScan 5^59^, Conserved Domain Database^60^, TMHMM 2.0^61^ and SignalP^35^. Putative virulence genes of *Py*. *insidiosum* were predicted based on a sequence homology search against the MvirDB database^25^. Adhesin-like proteins were predicted using FungalRV^62^. Selected proteins were aligned for sequence similarity and checked for protein architecture using MUSCLE^63^, NCBI BLAST and CD-search programs (https://blast.ncbi.nlm.nih.gov/Blast.cgi).

### Extraction of *Py*. *insidiosum* proteins

Ten small pieces (5 × 5 mm) of Sabouraud dextrose agar with actively growing *Py*. *insidiosum* (strain Pi-S), cut from a one-week-old colony, were transferred to two 500-ml Erlenmeyer flasks containing 100 ml of Sabouraud dextrose broth and incubated with shaking (150 rpm) for 10 days at 25 °C and 37 °C. Hyphae were harvested by filtration through Whatman filter paper No. 1 (pore size, 0.75 µm). The organism was ground in a precooled mortar, in the presence of liquid nitrogen. Ruptured hyphae were transferred to a 50-ml conical tube with 30 ml of cold sterile distilled water and mixed by inversion. The resulting cell lysate was centrifuged at 4,000 × g for 1 h (Beckman Coulter) to collect supernatant, which contained water-soluble proteins called soluble antigens from broken hyphae (SABHs)^64,65^. SABHs were concentrated 80-fold using an Amicon centrifugation tube (10,000 nominal-molecular-weight limit; Millipore), before storage at −20 °C. Overall, SABHs were prepared in four replicates (two each from *Py*. *insidiosum* grown at 25 °C and 37 °C).

### Preparation of tryptic-digested proteins

Ten micrograms of SABH was mixed with 2× protein loading buffer, containing 2× Laemmli sample buffer (Bio-Rad Laboratories, USA) and 2-mercaptoethanol, and denatured by heating at 65 °C for 10 min. The sample was subjected to one-dimensional SDS-PAGE, using 5% stacking and 12% resolving gels, and a mini-vertical electrophoresis system (Bio-Rad Laboratories, USA). The gels were stained with Coomassie brilliant blue G250. An individual lane of the SDS-PAGE gel was segmentally cut along its length into small pieces. To perform in-gel digestion, gel pieces were destained until colourless, using acetonitrile (50%) in 50 mM NH_4_HCO_3_. After the destaining solution had been removed, 10 mM dithiothreitol was added to the gel pieces, followed by incubation at 60 °C for 15 min. Proteins were alkylated using 55 mM iodoacetamide in 50 mM NH_4_HCO_3_ at room temperature for 30 min in the dark. Following the removal of all solution, the gel pieces were dehydrated by 100% ACN (Sigma-Aldrich, USA) and dried at room temperature. Protein digestion was performed overnight at 37 °C, using 0.1 mg/mL trypsin (Sigma-Aldrich, USA) in 50 mM ammonium bicarbonate. Peptides were extracted in 50% ACN. The resulting supernatant was transferred to microcentrifuge tubes and dried in a centrifugal concentrator (TOMY, Japan) at 45 °C.

### Liquid chromatography–mass spectrometric analysis

Each tryptic-digested fraction was resuspended in 0.1% formic acid and introduced to an Ultimate 3000 nano-LC system (Dionex, Surrey, UK). The separation was performed at a flow rate of 300 nL/min under a 45-min gradient. The Acclaim PepMap RSLC 75 μm × 15 cm nanoviper C18, 2-μm particle size, 100-Å pore size (Thermo Scientific, Waltham, MA), was used as a reverse-phase chromatography column. Mobile phase A was 2% (v/v) acetonitrile and 0.1% (v/v) formic acid in HPLC-grade water, and mobile phase B was 0.1% (v/v) formic acid in HPLC-grade acetonitrile. The eluate was sprayed to a MicroToF Q II mass spectrometer (Bruker, Bremen, Germany). Data were acquired using Hystar software (Bruker Daltonics, Germany). The survey scan mode covered the mass range of *m*/*z* 400–2500 and the MS/MS spectra covered the mass range of *m*/*z* 50–1500.

The ‘.d’ files from the microTOFQ II mass spectrometer were converted to ‘.mgf’ files by Compass DataAnalysis software (Bruker Daltonics, Germany) and used as queries for database sequence searches against the *Py*. *insidiosum* proteome^19^, using an in-house Mascot server (version 2.3.0; Matrix Science, USA). Trypsin was set as the enzyme. Only one missed cleavage site was allowed in the search. Variable modifications were set as carbamidomethyl (C) and oxidation (M). MS peptide and MS/MS tolerances were set at 0.6 Da and 0.8 Da, respectively. To reduce false-positive identification, only peptides with 95% confidence were reported in this study. Abundances of the proteins were calculated, based on the exponentially modified protein abundance index (emPAI)^66,67^. Each protein match was reported as (i) actual amount in emPAI units, (ii) ‘no emPAI’ (if the protein was detectable, but unquantifiable) or (iii) ‘n/a’ (if the protein was undetectable).

### Urease assay

A 5-mm-diameter Sabouraud dextrose agar plug with actively growing *Py*. *insidiosum* mycelia from three different isolates (Pi19, Pi20 and Pi-S) was transferred to a urease test tube (CLINAG, Thailand) and incubated at 37 °C for 7 days. Positivity for urease was identified if the urea-containing agar turned pink, while negativity was assigned if it remained yellow.

### Data availability

All data generated or analysed during this study are included in this published article (and its Supplementary Information files) and are available from the corresponding author on reasonable request. The DNA sequence of the putative urease gene (*Ure*1) present in the genome of *Py*. *insidiosum* (strain Pi-S) has been submitted to the DNA Data Bank of Japan database, under accession number LC317047.

## Electronic supplementary material

Supplementary information

Supplementary Table S1
