# Supplementary material for: Probing the Phylogenomics and Putative Pathogenicity Genes of Pythium insidiosum by Oomycete Genome Analyses
Source: Sci Rep. 2018 Mar 7;8:4135. doi: 10.1038/s41598-018-22540-1 (PMC5841299; doi:10.1038/s41598-018-22540-1)
Supplement: Supplementary file 1 — Supplementary information [file 41598_2018_22540_MOESM1_ESM.pdf]

Title: Probing the Phylogenomics and Putative Pathogenicity Genes of *Pythium insidiosum* by Oomycete Genome Analyses

Authors: Thidarat Rujirawat[1,2,3], Preecha Patumcharoenpol[4,5], Tassanee Lohnoo[2], Wanta Yingyong[2], Yothin Kumsang[2], Penpan Payattikul[2], Sithichoke Tangphatsornruang[6], Prapat Suriyaphol[7], Onrapak Reamtong[8], Gagan Garg[9], Weerayuth Kittichotirat[5*], Theerapong Krajaejun[1*]

Affiliations: [1]Department of Pathology, Faculty of Medicine, Ramathibodi Hospital, Mahidol University, Bangkok, Thailand; [2]Research Center, Faculty of Medicine, Ramathibodi Hospital, Mahidol University, Bangkok, Thailand; [3]Molecular Medicine Program, Multidisciplinary Unit, Faculty of Science, Mahidol University, Bangkok, Thailand; [4]Department of Biomedical Informatics, University of Arkansas for Medical Sciences, Little Rock, Arkansas 72205, USA; [5]Systems Biology and Bioinformatics Research Group, Pilot Plant Development and Training Institute, King Mongkut's University of Technology Thonburi, Bangkok, Thailand; [6]Genomic Research Laboratory, National Center for Genetic Engineering and Biotechnology, National Science and Technology Development Agency, Pathumthani, Thailand; [7]Bioinformatics and Data Management for Research, Office for Research and Development, Faculty of Medicine, Siriraj Hospital, Mahidol University, Bangkok, Thailand; [8]Department of Molecular Tropical Medicine and Genetics, Faculty of Tropical Medicine, Mahidol University, Bangkok, Thailand; [9]CSIRO Agriculture and Food, Centre for Environment and Life Sciences, Floreat, WA, Australia

***Corresponding authors:** Theerapong Krajaejun, mr_en@hotmail.com; Weerayuth Kittichotirat, weerayuth.kit@kmutt.ac.th

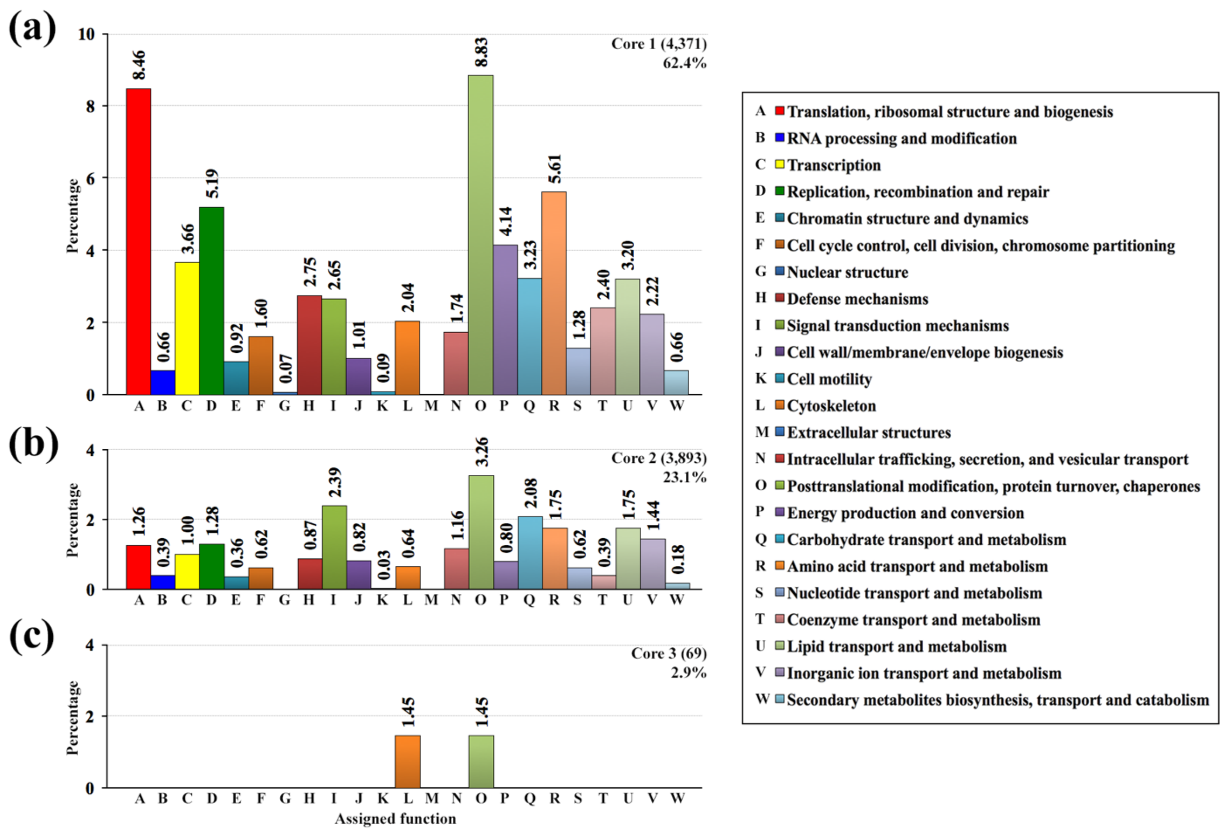

Supplementary Figure S1. Twenty-three Clusters of Orthologous Groups (COGs) assigned to: (**a**) 62.4% of 4,371 Core 1 gene clusters (belonging to oomycetes and diatoms), (**b**) 23.1% of 3,893 Core 2 gene clusters (belonging to oomycetes) and (**c**) 2.9% of 69 Core 3 gene clusters (belonging to the genus *Pythium*). At the top of each bar is the percentage of Core 1, 2 or 3 gene clusters that matched each COG. A description of each COG (as indicated by colours and letters A to W) is shown in the box.

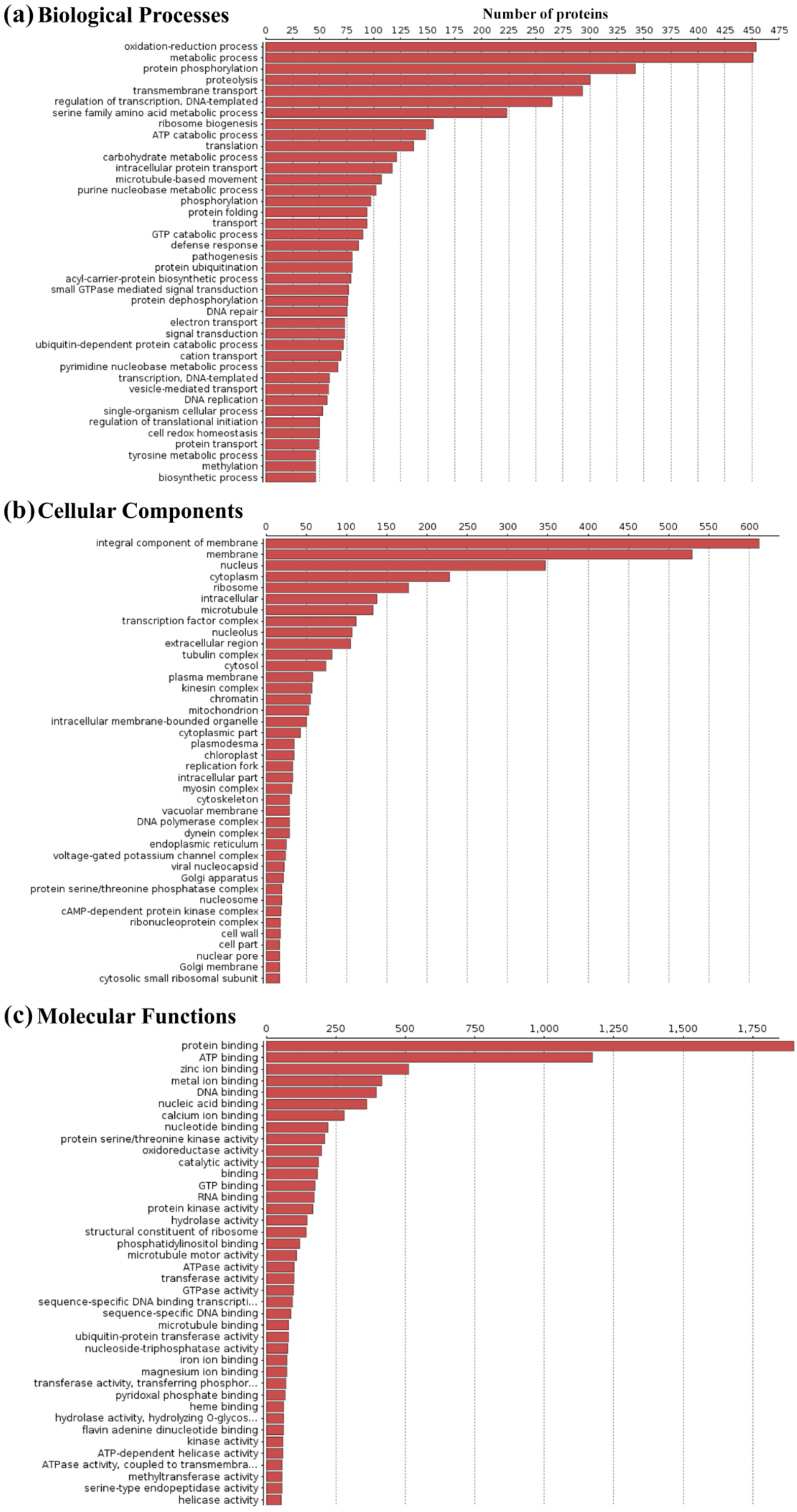

Supplementary Figure S2. The top 40 Gene Ontology (GO) terms assigned to the predicted proteins of *Pythium insidiosum*, for Biological Processes (**a**), Cellular Components (**b**) and Molecular Functions (**c**).

Supplementary Table S1. The core genes (n = 2,073) identified in all 20 genomes of oomycetes (supplementary file).

Supplementary Table S2. The enzyme commission (EC) classes and subclasses of predicted enzymes identified in the genome of *Pythium insidiosum*. The number of matched proteins in each EC subclass is shown.

EC class / subclass	Number of proteins
**EC class-1: Oxidoreductases**	
EC:1.1 : Acting on the CH-OH group of donors	49
EC:1.2 : Acting on the aldehyde or oxo group of donors	15
EC:1.3 : Acting on the CH-CH group of donors	30
EC:1.4 : Acting on the CH-NH_2 group of donors	4
EC:1.5 : Acting on the CH-NH group of donors	14
EC:1.6 : Acting on NADH or NADPH	8
EC:1.7 : Acting on other nitrogenous compounds as donors	1
EC:1.8 : Acting on a sulfur group of donors	12
EC:1.9 : Acting on a heme group of donors	2
EC:1.10 : Acting on diphenols and related substances as donors	3
EC:1.11 : Acting on a peroxide as acceptor	21
EC:1.13 : Acting on single donors with O_2 as oxidant	6
EC:1.14 : Acting on paired donors, with O_2 as oxidant	20
EC:1.15 : Acting on superoxide as acceptor	4
EC:1.16 : Oxidizing metal ions	1
EC:1.17 : Acting on CH or CH_2 groups	3
**EC class-2: Transferases**	
EC:2.1 : Transferring one-carbon groups	43
EC:2.2 : Transferring aldehyde or ketonic groups	6
EC:2.3 : Acyltransferases	44
EC:2.4 : Glycosyltransferases	38
EC:2.5 : Transferring alkyl or aryl groups, other than methyl groups	18
EC:2.6 : Transferring nitrogenous groups	13
EC:2.7 : Transferring phosphorus-containing groups	275
EC:2.8 : Transferring sulfur-containing groups	5
**EC class-3: Hydrolases**	
EC:3.1 : Acting on ester bonds	126
EC:3.2 : Glycosylases	4
EC:3.3 : Acting on ether bonds	2
EC:3.4 : Acting on peptide bonds (peptidases)	88
EC:3.5 : Acting on carbon-nitrogen bonds, other than peptide bonds	27
EC:3.6 : Acting on acid anhydrides	177
EC:3.7 : Acting on carbon-carbon bonds	1
**EC class-4: Lyases**	
EC:4.1 : Carbon-carbon lyases	22
EC:4.2 : Carbon-oxygen lyases	32
EC:4.3 : Carbon-nitrogen lyases	10
EC:4.4 : Carbon-sulfur lyases	4
**EC class-5: Isomerases**	
EC:5.1 : Racemases and epimerases	1
EC:5.2 : cis-trans-Isomerases	24
EC:5.3 : Intramolecular oxidoreductases	13
EC:5.4 : Intramolecular transferases	14
EC:5.5 : Intramolecular lyases	1
EC:5.99 : Other isomerases	12
**EC class-6: Ligases**	
EC:6.1 : Forming carbon-oxygen bonds	51
EC:6.2 : Forming carbon-sulfur bonds	7
EC:6.3 : Forming carbon-nitrogen bonds	33
EC:6.4 : Forming carbon-carbon bonds	6
EC:6.5 : Forming phosphoric-ester bonds	6
**Total**	**1296**

Supplementary Table S3. Description of functional domains, identified in 10,242 predicted proteins of *Pythium insidiosum*. The top 40 domains with the highest frequency are shown.

InterPro number	Domain	Frequency of domains
IPR027417	P-loop containing nucleoside triphosphate hydrolase	726
IPR020683	Ankyrin repeat-containing domain	670
IPR001680	WD40 repeat	619
IPR000719	Protein kinase domain	575
IPR002110	Ankyrin repeat	479
IPR017986	WD40-repeat-containing domain	446
IPR011009	Protein kinase-like domain	393
IPR002048	EF-hand domain	373
IPR013083	Zinc finger, RING/FYVE/PHD-type	356
IPR011992	EF-hand domain pair	326
IPR001849	Pleckstrin homology domain	325
IPR001841	Zinc finger, RING-type	298
IPR016024	Armadillo-type fold	291
IPR001202	WW domain	286
IPR001683	Phox homologous domain	285
IPR015943	WD40/YVTN repeat-like-containing domain	276
IPR000048	IQ motif, EF-hand binding site	260
IPR001752	Kinesin, motor domain	256
IPR000504	RNA recognition motif domain	255
IPR000008	C2 domain	251
IPR001650	Helicase, C-terminal	241
IPR001623	DnaJ domain	240
IPR019734	Tetratricopeptide repeat	227
IPR011990	Tetratricopeptide-like helical	226
IPR016040	NAD(P)-binding domain	223
IPR011989	Armadillo-like helical	218
IPR000595	Cyclic nucleotide-binding domain	209
IPR002290	Serine/threonine-dual specificity protein kinase	207
IPR000306	FYVE zinc finger	200
IPR001478	PDZ domain	195
IPR029058	Alpha/Beta hydrolase fold	192
IPR011011	Zinc finger, FYVE/PHD-type	190
IPR011993	Pleckstrin homology-like domain	185
IPR014001	Helicase, superfamily 1/2, ATP-binding domain	181
IPR000569	HECT	177
IPR018247	EF-Hand 1, calcium-binding site	171
IPR003593	AAA+ ATPase domain	167
IPR012336	Thioredoxin-like fold	152
IPR016196	Major facilitator superfamily domain	152
IPR003439	ABC transporter-like	151

Supplementary Table S4. The top 30 proteins of *Pythium insidiosum* that exhibit at least twofold up- and downregulation, upon exposure to an increase in temperature from 25°C to 37ºC. The identification number, description, LC-MS/MS-derived protein abundance (emPAI value; see the methods) and fold change in expression of each protein from the organism grown at 25ºC and 37ºC are shown. 'Up' and 'Down' indicate up- and downregulated proteins, respectively.

No	Protein ID	Description	25℃ Growth		37℃ Growth		Up/Down regulated	Fold changed
			Average emPAI value	SD	Average emPAI value	SD		
1	PINS01640001A	Cyclophilin A	1.71	1.07	76.22	43.15	Up	44.7
2	PINS01990007A	Peroxiredoxin-2	0.68	0.21	10.05	-	Up	14.9
3	PINS00240001A	Inositol-3-phosphate synthase	0.06	-	0.81	0.08	Up	13.4
4	PINS00240030A	Membrane alanine aminopeptidase	0.08	0.02	0.74	0.07	Up	9.9
5	PINS00860003A	Proteasome subunit beta type-6	0.15	0.01	1.41	0.23	Up	9.7
6	PINS00010113A	Proteasome subunit alpha type-6	0.13	0.01	0.94	0.48	Up	7.5
7	PINS00530045C	Hit domain-containing protein	0.11	-	0.75	0.13	Up	6.8
8	PINS00550024A	5-methyltetrahydropteroyltriglutamate-homocysteine s-methyltransferase	0.85	0.05	5.76	0.53	Up	6.8
9	PINS00400073A	Ornithine carbamoyltransferase	0.09	-	0.61	0.46	Up	6.7
10	PINS01180004A	Cytochrome c	6.12	6.09	38.77	13.44	Up	6.3
11	PINS04140003A	Ornithine carbamoyltransferase	0.09	-	0.53	0.35	Up	5.9
12	PINS00560003A	Carbohydrate esterase	1.15	0.08	6.55	3.78	Up	5.7
13	PINS01150012B	Dihydroorotase, homodimeric type	0.09	0.04	0.48	0.12	Up	5.3
14	PINS01150027A	Heat shock 70 kDa protein, mitochondrial precursor	0.18	0.03	0.92	0.25	Up	5.1
15	PINS01410019A	Hypothetical protein	0.06	-	0.31	0.26	Up	5.1
16	PINS00710026A	Urocanate hydratase	0.16	0.01	0.76	0.06	Up	4.9
17	PINS01380017B	Dihydrolipoyl dehydrogenase 1, mitochondrial precursor	0.04	0.01	0.17	0.14	Up	4.9
18	PINS00390012A	Protein disulfide-isomerase	0.13	-	0.62	0.27	Up	4.8
19	PINS00630019A	Hypothetical protein	1.22	0.66	5.56	1.70	Up	4.6
20	PINS00170026C	Fatty acid synthase subunit alpha	0.01	-	0.05	0.01	Up	4.5
21	PINS00910027B	Hypothetical protein	0.03	0.01	0.11	-	Up	4.4
22	PINS00620003C	Hypothetical protein	0.02	0.01	0.06	-	Up	4.0
23	PINS00430026C	Protein kinase	0.10	-	0.39	-	Up	3.9
24	PINS00590023A	Hypoxanthine phosphoribosyltransferase	0.42	0.41	1.58	0.23	Up	3.8
25	PINS01930014A	2-amino-3-ketobutyrate coenzyme A ligase	0.08	0.01	0.28	0.06	Up	3.7
26	PINS00450059A	Methylmalonate-semialdehyde dehydrogenase	0.30	0.04	1.03	0.08	Up	3.4
27	PINS01840005B	60s ribosomal protein L18a	0.18	0.01	0.59	0.17	Up	3.4
28	PINS00970028B	Argininosuccinate synthase	0.04	-	0.13	-	Up	3.3
29	PINS00260047A	Eukaryotic translation initiation factor 6	0.14	0.01	0.43	-	Up	3.2
30	PINS00350004A	Hypothetical protein	0.13	0.04	0.40	0.28	Up	3.2
31	PINS00200016A	6-phosphogluconate dehydrogenase	8.01	0.88	0.20	0.10	Down	(40.0)
32	PINS01370001A	Hypothetical protein	10.78	6.18	0.50	0.28	Down	(21.8)
33	PINS00120035C	Glucokinase	0.40	0.11	0.04	-	Down	(9.9)
34	PINS01350003C	Hypothetical protein	0.31	0.01	0.04	-	Down	(7.8)
35	PINS00720029C	Enolase	1.04	0.04	0.16	0.11	Down	(6.5)
36	PINS00060066A	Alpha-soluble nsf attachment protein	0.27	0.06	0.05	-	Down	(5.4)
37	PINS00640043C	Endo-1,3-beta-glucanase	2.02	0.60	0.41	-	Down	(4.9)
38	PINS02480006A	Glyceraldehyde-3-phosphate dehydrogenase	16.27	1.07	3.45	0.55	Down	(4.7)
39	PINS02480014A	Glyceraldehyde-3-phosphate dehydrogenase	12.16	0.66	2.74	0.46	Down	(4.4)
40	PINS00100075A	Hypothetical protein	9.39	4.16	2.15	-	Down	(4.4)
41	PINS01480032A	Glutamate decarboxylase	0.77	0.04	0.19	0.09	Down	(4.2)
42	PINS01630011B	Annexin family	2.32	0.37	0.59	0.65	Down	(3.9)
43	PINS01820013C	Glutamate decarboxylase	0.29	0.01	0.08	0.02	Down	(3.9)
44	PINS00120088A	Hypothetical protein	0.40	0.11	0.11	0.05	Down	(3.8)
45	PINS00020126A	Ornithine aminotransferase, mitochondrial	3.50	0.73	0.94	0.18	Down	(3.7)
46	PINS00020067B	Hypothetical protein	0.11	0.01	0.03	0.03	Down	(3.7)
47	PINS00150113C	Hypothetical protein	0.47	0.03	0.13	-	Down	(3.6)
48	PINS01380002A	Hypothetical protein	0.36	0.30	0.11	0.05	Down	(3.4)
49	PINS00210018A	Spermidine synthase	0.72	0.08	0.21	-	Down	(3.4)
50	PINS00050072A	Fructose-1,6-bisphosphatase	2.56	0.65	0.79	0.85	Down	(3.2)
51	PINS01460014A	Cysteine-rich protein	0.78	0.29	0.25	0.23	Down	(3.1)
52	PINS00430046C	Tnxb	0.03	-	0.01	-	Down	(3.0)
53	PINS01960012C	Hypothetical protein	0.03	-	0.01	-	Down	(3.0)
54	PINS01930007A	Enolase	7.48	3.66	2.52	0.60	Down	(3.0)
55	PINS00020065A	Chaperonin grol	5.82	0.59	1.99	0.23	Down	(2.9)
56	PINS03670006A	Hypothetical protein	0.60	0.44	0.22	0.07	Down	(2.7)
57	PINS00770019C	Hypothetical protein	1.53	0.01	0.57	0.41	Down	(2.7)
58	PINS00500027C	Fructose-bisphosphate aldolase	1.71	0.25	0.65	0.20	Down	(2.6)
59	PINS00100073B	DNA polymerase subunit alpha B	0.44	0.13	0.17	-	Down	(2.6)
60	PINS00500049A	Putative alpha amylase	1.73	0.08	0.70	0.40	Down	(2.5)
